# Geocoding Error, Spatial Uncertainty, and Implications for Exposure Assessment and Environmental Epidemiology

**DOI:** 10.3390/ijerph17165845

**Published:** 2020-08-12

**Authors:** Ellen J. Kinnee, Sheila Tripathy, Leah Schinasi, Jessie L. C. Shmool, Perry E. Sheffield, Fernando Holguin, Jane E. Clougherty

**Affiliations:** 1 University Center for Social and Urban Research, University of Pittsburgh, Pittsburgh, PA 15260, USA; 2 Department of Environmental and Occupational Health, Drexel University Dornsife School of Public Health, Philadelphia, PA 19104, USA; sheila.tripathy@gmail.com (S.T.); lhs36@drexel.edu (L.S.); jec373@drexel.edu (J.E.C.); 3 Drexel University Urban Health Collaborative (UHC), Drexel University Dornsife School of Public Health, Philadelphia, PA 19104, USA; 4 Department of Environmental and Occupational Health, University of Pittsburgh Graduate School of Public Health, Pittsburgh, PA 15260, USA; jlcshmool@gmail.com; 5 Environmental Medicine and Public Health and Pediatrics, Icahn School of Medicine at Mount Sinai, New York, NY 10029, USA; perry.sheffield@mssm.edu; 6 Department of Medicine, University of Colorado School of Medicine, Aurora, CO 80045, USA; Fernando.holguin@cuanschutz.edu

**Keywords:** geocoding error, exposure misclassification, geographic information systems (GIS), spatial analysis, spatial uncertainty, urban epidemiology

## Abstract

Although environmental epidemiology studies often rely on geocoding procedures in the process of assigning spatial exposure estimates, geocoding methods are not commonly reported, nor are consequent errors in exposure assignment explored. Geocoding methods differ in accuracy, however, and, given the increasing refinement of available exposure models for air pollution and other exposures, geocoding error may account for an increasingly larger proportion of exposure misclassification. We used residential addresses from a reasonably large, dense dataset of asthma emergency department visits from all New York City hospitals (*n* = 21,183; 26.9 addresses/km^2^), and geocoded each using three methods (Address Point, Street Segment, Parcel Centroid). We compared missingness and spatial patterning therein, quantified distance and directional errors, and quantified impacts on pollution exposure estimates and assignment to Census areas for sociodemographic characterization. Parcel Centroids had the highest overall missingness rate (38.1%, Address Point = 9.6%, Street Segment = 6.1%), and spatial clustering in missingness was significant for all methods, though its spatial patterns differed. Street Segment geocodes had the largest mean distance error (µ = 29.2 (SD = 26.2) m; vs. µ = 15.9 (SD = 17.7) m for Parcel Centroids), and the strongest spatial patterns therein. We found substantial over- and under-estimation of pollution exposures, with greater error for higher pollutant concentrations, but minimal impact on Census area assignment. Finally, we developed surfaces of spatial patterns in errors in order to identify locations in the study area where exposures may be over-/under-estimated. Our observations provide insights towards refining geocoding methods for epidemiology, and suggest methods for quantifying and interpreting geocoding error with respect to exposure misclassification, towards understanding potential impacts on health effect estimates.

## 1. Introduction

A growing number of population-based studies rely on geocoding (i.e., assignment of x and y coordinates (latitude and longitude)) to assign spatial exposure estimates [1,2,3,4]. Despite this tremendous reliance on geocoding methods, relatively few epidemiologic studies examine and report geocoding errors in substantive detail. Geocoding methods differ in accuracy, however, for many reasons, including differing resolutions of the underlying reference (geolocator) data, and methods for linking addresses to reference data. The resultant spatial uncertainty is often of unknown magnitude and direction, as are its impacts on exposure misclassification and health effect estimates. Finally, in recent years, there has been great emphasis on the development of fine-scale exposure models, particularly for urban air pollution [5,6,7]; there has not been corresponding attention paid to geocoding methods, which can induce errors of several hundred meters [8,9,10].

Many studies in the geography literature have evaluated issues in geocoding, including missingness (i.e., unmatched addresses) and positional accuracy (i.e., accuracy of x,y assignment, in distance or direction) [11,12,13,14]. We found more geography studies of distance error (i.e., Euclidean distance displacement from reference point) [8,11,15,16] than of directional error (i.e., cardinal direction of displacement) [8], and only a few discussing or quantifying spatial clustering in error [17,18] or missingness [14]. Most studies were performed in less-dense areas [17,19,20], or relied on smaller datasets [9,21,22], limiting their ability to examine systematic spatial clustering in errors and its consequent impacts on exposure assignment. Geocoding errors, however, are generally not random [13,23,24], and the resultant clustering, and its implications for exposure assignment or effect estimates, have been rarely explored [14,25]. Only a few epidemiologic studies have imputed exposures for missing (unmatched) geocodes [9], or compared effect estimates across strata defined by accuracy or missingness [26].

Of particular importance for environmental epidemiology is whether geocoding errors vary with (i.e., are differential by) either exposures or outcomes [27], and only a few studies have assessed the impacts of positional error on exposure misclassification or effect estimates [16,22,28]. For example, Goldman et al. [29] found that spatial misalignment reduced risk ratios for air pollution by 16% to 68%, depending on the pollutant’s spatial heterogeneity. Zandbergen et al. (2012) [20] found differential error by race in assigning Census-based sociodemographic indicators, as minorities were more likely to live in higher-density urban areas, where areal units were smaller and more prone to mis-assignment.

In exposure assessment and epidemiology, there is substantial risk of error propagation in comparing multiple layers of spatial data (e.g., linking geocodes to pollution surfaces), and thus a need to assess the error that is attributable to each [25,30]. To date, however, there are few examples of practical methods for reporting and incorporating geocoding error into epidemiology [25,31,32,33]. Schinasi 2018 [1] found that 62% of articles using EHR data linked with geospatial data did not report measures of uncertainty owing to low match rates or geocoding error. It thus remains unclear in most studies whether errors varied across space or exposure intensity, how geocoding errors influenced exposure estimates or spatial confounding among exposures, and whether and how positional errors may influence health effect estimates [25].

Here, we examine spatial patterns in geocoding error and exposure misclassification using a reasonably large, dense dataset of addresses from pediatric hospitals records in New York City (NYC). We compare three common geocoding methods (Address Point, Parcel Centroid, Street Segment), focusing on geocoding error as it relates to fine-scale air pollution and Census sociodemographic assignments. We examine missingness and spatial patterns therein, quantify positional errors (in both distance and direction) and likewise examine spatial patterns therein, and examine the impacts of positional error on environmental and social exposure estimates, testing whether those errors vary along the exposure gradient. Finally, we generate spatial surfaces of distance errors (i.e., uncertainty surfaces) to depict spatial patterns in error, which may be overlaid with cohort distributions, in order to produce location-specific uncertainty estimates. Though we developed these methods using a large hospitals-based dataset, they are applicable to any set of cohort addresses. We aim to highlight the importance of evaluating geocoding error for large urban datasets in order to reduce its impact on exposure assignment and, consequently, epidemiologic effect estimates.

## 2. Materials and Methods

### 2.1. Datasets

#### 2.1.1. Residential Address Data

**Figure 1:**
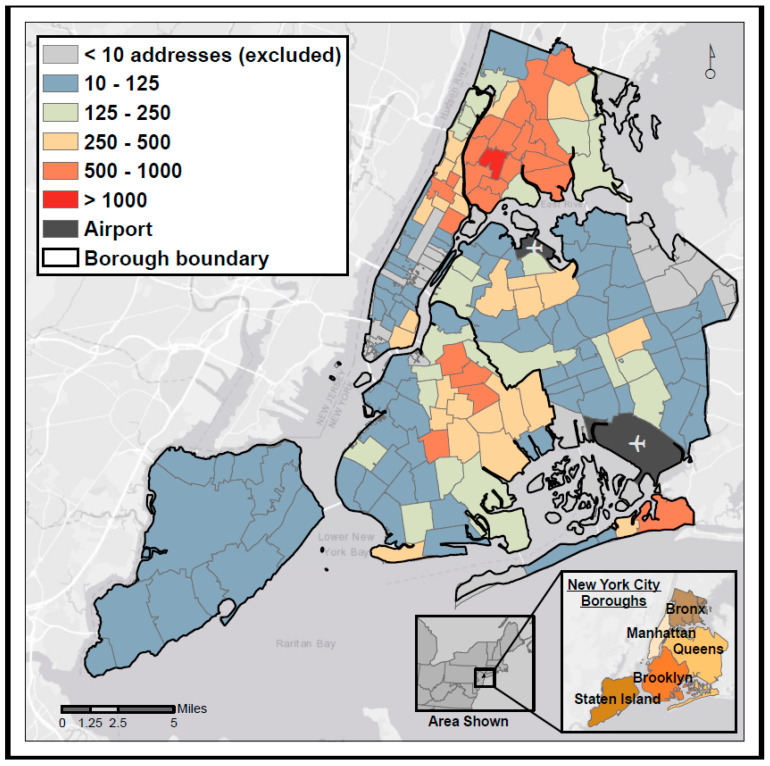
Spatial distribution of the 21,183 residential addresses used in this analysis, aggregated to ZIP Code.

Residential addresses were drawn from NYC hospital emergency department (ED) data (2005–2011), used for an epidemiologic study on ozone and asthma exacerbations [34], obtained from the New York Statewide Planning and Research Cooperative System (SPARCS) (*n* = 35,907). Cases were drawn from all 157 populated 5-digit ZIP Codes in NYC, though the number of addresses per ZIP Code varied widely (Figure 1). All comparisons reported here include only those addresses that could be geocoded using all three locators (*n* = 21,183), producing a conservative analysis (Appendix A: Detailed Geocoding Methods).

#### 2.1.2. Air Pollution Data

Spatial surfaces for annual-average nitrogen dioxide (NO_2_), particulate matter (PM_2.5_) and summer ozone (O_3_) concentrations were provided by New York City Community Air Survey [35,36,37] (NYCCAS), a surveillance initiative by the New York City Department of Health and Mental Hygiene (DOHMH). Briefly, pollution was sampled at 150 locations citywide for two years, December 2008–November 2010. Land Use Regression (LUR) models—a multivariate modeling approach which describes intra-urban spatial variance in pollution as a function of GIS-based indicators of local pollution sources and land use variability (e.g., traffic and diesel traffic density, buildings density, proximity to industry and industrial emissions) [38]—were developed to identify key pollution sources, and to estimate associations between measured concentrations and GIS-based source indicators. LUR models were used to estimate pollution concentrations at the centroid of each 100 m × 100 m cell across NYC. We focus this analysis on NO_2_, which was monitored using passive Ogawa badges at two-week intervals, and was largely predicted by traffic and buildings density in NYCCAS LUR models [35,37]. NO_2_ shows greater fine-scale spatial variance, relative to other pollutants [35,37], with complex patterning by socioeconomic position [39]; as such, fine-scale geocoding error may plausibly have a greater impact on NO_2_ estimation than on other pollutants.

#### 2.1.3. Socioeconomic Position Data

Poverty rate was selected as one key indicator of socioeconomic position (SEP), following prior geographic research finding comparable results in spatial patterning using poverty alone or more complex multivariable indices [40,41], and for comparability to larger efforts to assess socioeconomic patterns in health across NY State [42].

Poverty rate was defined as percent of the population living below the federal poverty level. Data were drawn from the American Communities Survey (ACS) five-year estimates for 2008–2012 [43], using year 2010 CT boundaries. The U.S. Census 2010 boundary files for multiple administrative areas (Census Tract, Block Group, Block) were used to assess the influence of the geocoding method on point-in-polygon assignments to Census areas.

### 2.2. Geocoding Methods

We applied a multi-step composite geocoding process, first cleaning and standardizing address formats and then separately geocoding all addresses using each of the three geolocator reference datasets: Address Points, Street Segments and tax parcels, as detailed elsewhere [44]. Briefly, the *Address Point* locator is based on discrete United State Postal Service (USPS) delivery point locations associated with established physical buildings. The *Street Segment* locator assigns addresses to a point location along the street by interpolating the position of the address number along the known range of addresses on that segment. The *Parcel Centroid* locator matches addresses to the centroids of the corresponding NYC tax parcels, which range in size from an average of 474.1 m^2^ in Brooklyn, to 981.7 m^2^ in Manhattan (multi-story, multi-family buildings in larger parcels). In keeping with our goal of assessing customary practices, we retained the default settings for the three locator styles (ArcGIS™ 10.5 (ESRI, Redlands, CA, USA)). The parameter settings and reference data sources for all individual locators are shown in Appendix A: Detailed Geocoding Methods.

The Address Point and Parcel Centroid locators are designed to locate an address within its corresponding building footprint or tax parcel boundary; in contrast with this, Street Segment locators interpolate along a street centerline, and rely on a default offset to position points closer to building centroids. Our Street Segment locator uses a default side offset of 20 feet (6.10 m), applied perpendicular to the street centerline. We performed a sensitivity analysis to evaluate how fixed-side offsets affect geocoding accuracy for street segment geocodes (Figure S1 in Supplemental Materials), and found that changing the offset distance introduced both small increases and decreases in positional accuracy (<1 m) that were inconsistent and unpredictable across the domain. Several studies have found that any changes to side offset distance result in little to no improvement in measured distance errors. [8,10,45]

### 2.3. Geocoding Error Measurement

We measured three dimensions of geocoding error—(1) *missingness*, (i.e., the percent of non-geocodeable addresses within a given area) and spatial patterning therein, (2) *distance error* (i.e., Euclidean distance displacement from a geocoded point (here, using Street Segment or Parcel Centroid locators) to its ‘true’ reference location (here, using Address Point as the reference)), and (3) *directional error* (i.e., cardinal direction of displacement from a ‘true’ reference location to its alternative geocoded location). Additionally, we quantified the effect of geocoding error on the assignment of air pollution exposure estimates and the Census SEP indicator.

We first ran the ArcGIS™ Incremental Spatial Autocorrelation tool to identify the critical distances within which spatial clustering is apparent; these distances reflect the underlying scale of the relevant spatial processes, and are used here as the fixed-distance band or threshold distances for clustering analyses. Spatial clusters in each type of error (spatial errors in missingness, distance and direction, and errors in estimated pollution exposures) were then identified using the Cluster and Outlier Analysis tool in ArcGIS™ 10.5 [46,47]. This tool calculates an Anselin Local Moran’s *I* index [46], a z-score (a relative Moran’s *I* value for each observation in the dataset, centered at mean = 0 and standard deviation = 1), and a cluster-type code for each feature. A high positive z-score means the surrounding points have similar high or low values; where the *p*-value is <0.05, there is a less than 5% probability of observing similar high or low values in the surrounding areas, assuming that the null hypothesis (no clustering) is true. Observing this statistical significance suggests that there is a true underlying spatial process, and the feature is considered to belong to a true ‘high’ or ‘low’ cluster.

#### 2.3.1. Missingness and Spatial Patterning in Missingness

Missingness rates are defined as the percent of addresses not successfully geocoded within a given area. As such, in order to estimate and compare missingness across space, we needed to create rates for small areas. Because, by definition, we do not know the locations of the unsuccessfully geocoded addresses in our dataset (we could not assign them to an x,y), we cannot assign them to a geographic unit (e.g., Census tract)—with the exception of the ZIP code, which is a separate field in the address database. As such, we calculated, for each ZIP code, the percent of the addresses in it which were successfully geocoded, and compared missingness (the inability to match addresses to x,y locations) across the 157 NYC ZIP Codes with more than 10 records in our dataset. Missingness was quantified as the percent of all addresses within each ZIP Code that were *not* successfully geocoded (=100%—match rate), using each of the three geocoding methods (Address Point, Street Segment, Parcel Centroid), respectively.

#### 2.3.2. Distance Error

All analyses of distance error—Euclidean distance from a geocoded point to its ‘true’ reference location—were performed at the address level. Because we anticipated that the Address Point locator would confer the highest level of validity among the three methods, we used this method as the reference, to which we compared Street Segment and Parcel Centroid results. This expectation follows on prior research documenting fewer false positive matches (i.e., geocoding a building which does not exist) using Address Points, relative to Street Segment interpolation [9,15]. Further, Address Point data may offer greater consistency than tax parcel data which, maintained for tax purposes, often lists the mailing address of the building owner, rather than the building’s physical location; in cities like NYC, with very high rental rates (51%) [48], this error could impact a large percentage of residential geocodes.

Distance errors were calculated as Euclidian distance in meters from each Street Segment or Parcel Centroid geocode to the corresponding Address Point (reference) geocode.

#### 2.3.3. Directional Error

Directional error was defined as the compass angle (clockwise from due North) of the straight line drawn from each Address Point to its corresponding Street Segment and Parcel Centroid geocodes, indicating the angle of displacement from the ‘true’ (Address Point) location. Specialized rose plots were generated using the Polar Plots Extension for GIS [49] in order to display the frequency of directional errors by compass direction divided into 5-degree bins, resulting in 72 cardinal classes. Although there is some loss of information in converting continuous angular data into cardinal classes, the method helps to determine whether error is uniformly distributed around the circle, or has a common mean direction [50]. Multiple statistical measures have been designed to test circular uniformity (i.e., distribution of directions) [51], and we calculated mean vector direction in order to determine the primary directional orientation of errors.

### 2.4. Exposure Assignment Impacts

#### 2.4.1. Air Pollution Exposure Assignment

Individual-level near-residence air pollution exposures were estimated, using each set of geocodes, as the mean NYCCAS concentration for each pollutant within 300 m of the geocoded residence (Figure S2, Supplemental Material.doc); this buffer size was previously validated for exposure assignment in epidemiologic studies using the NYCCAS pollution surfaces, as described in Ross (2013) [52]. Given NYC’s many large multi-unit residential buildings, and the likelihood that some individuals will go to the emergency department repeatedly, we checked for repeated addresses in our data, which accounted for only 799 visits (2.2%) in total (Figure S3, Supplemental Material.doc). We retained repeat addresses in all analysis, as these would be present in most urban datasets, and excluding them would under-represent the most densely populated areas of the city. We calculated percent over- and under-estimates using the concentrations predicted using the Street Segment and Parcel Centroid locators, as a function of those predicted using the Address Point locator, and compared these percentages across pollutants, by geocoding method, using t-tests, after testing for normality, in SAS v 9.4 (SAS Institute, Cary, NC, USA).

#### 2.4.2. Socioeconomic Position Exposure Assignment

We developed a measure of predicted error in assignment to Census areas by overlaying each of the three geocoded points per address onto each of three different U.S. Census boundaries (Tract, Block Group, Block), which are all commonly used to derive SEP indicators using the American Community Survey (ACS) and other sociodemographic data. We calculated the percentage of addresses which would have been assigned to a neighboring Census area using an alternative geocoding method, and assessed the consequent effects of these locational errors on changing the assignment of SEP indicators.

#### 2.4.3. Spatial Clustering in NO_2_ Exposure Estimates

Using the Address Point geocodes as our reference, we subtracted exposure estimates derived using this method from those using the two alternative methods (Street Segment and Parcel Centroid), and examined the resultant differences. Spatial clustering in high (over-estimated) and low (under-estimated) NO_2_ exposure estimates was identified using the local Moran’s *I* statistic, as described in Section 2.3. These clusters were then overlaid with tract-level poverty level to assess differential misclassification in pollution exposure estimates by SEP.

#### 2.4.4. Differential Error in Air Pollution Exposure Estimates

Bland–Altman plots, which depict the difference between two values as a function of their mean [53,54], were used to examine differential error due to geocoding method in case-level exposure estimates for three air pollutants (NO_2_, PM_2.5_, O_3_). The 95% ‘limits of agreement’ were generated using mean difference between each pair of geocodes (i.e., bias) ± 1.96 x standard deviation (SD) [55]. These limits include 95% of the differences between the pair [56]. Any points falling outside of these limits are then mapped to identify spatial distributions and clustering in errors.

#### 2.4.5. Spatial Uncertainty Surfaces

To identify areas with more or less locational uncertainty within the study domain, we used the IDW (inverse-distance weighting) tool (ArcGIS™ 10.5) to produce interpolated raster surfaces of measured distance errors. To demonstrate the utility of the uncertainty surfaces in assessing potential error in exposure assignments, and as a type of cross-validation, we overlaid geocoded addresses onto the uncertainty surfaces, to compare expected vs. measured distance errors. This approach would enable researchers to identify addresses located within relatively high- or low-accuracy areas for each method, or to assess potential errors that may be induced using an alternative geocoding method.

## 3. Results

### 3.1. Missingness

The overall missingness rate (percent of addresses not successfully geocoded) was highest for Parcel Centroids (38.1%), compared to 9.6% for Address Points, and 6.1% for Street Segments.

**Figure 2:**
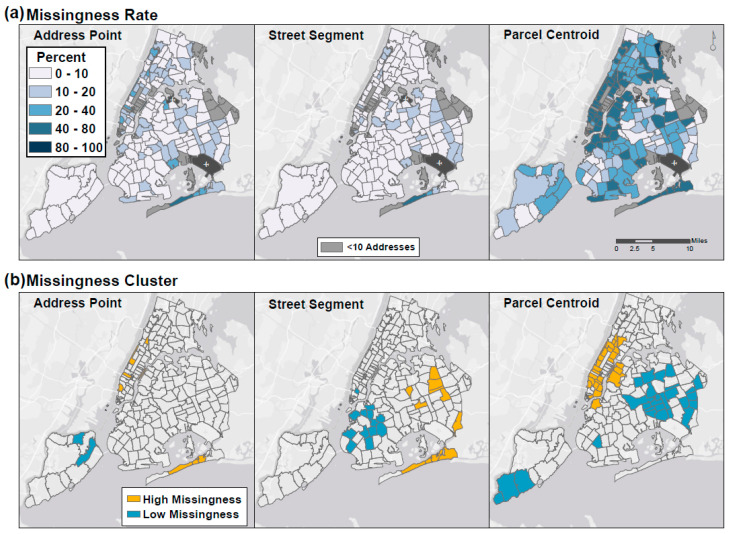
Spatial patterns of missingness by 5-digit ZIP Code. Maps of (**a**) rates of missingness and (**b**) statistical clusters of high (neighboring ZIP Codes have similarly high rates) and low (neighboring ZIP Codes have similarly low rates) levels of missingness.

To understand spatial patterns and clustering in missingness, we calculated missingness rates by ZIP code, which were highest, on average, using the Parcel Centroid locator (35.0% (SD = 8.4%)), compared to 9.9% using either the Address Point or Street Segment locator (SD = 8.4% and 6.0%, respectively), (Table S1 in Supplemental Materials). We found distinct spatial patterns and clustering in missingness, which varied by method (Figure 2). Local Moran’s *I* statistics indicated clustering at the 95% confidence level in missingness for each method; Address Point missingness displayed the least clustering.

### 3.2. Distance Error

Distance error—defined here as Euclidian distance from the Address Point—was greater, on average, for Street Segment (µ = 29.2 m (SD = 26.2 m); range = 0.94–1038.9 m) than for Parcel Centroid geocodes (µ = 15.9 m (SD = 15.7 m); range = 0.3–287.3 m). Importantly, there were no distance errors of zero; each of the three methods generated a different point location for all 21,183 addresses.

**Figure 3:**
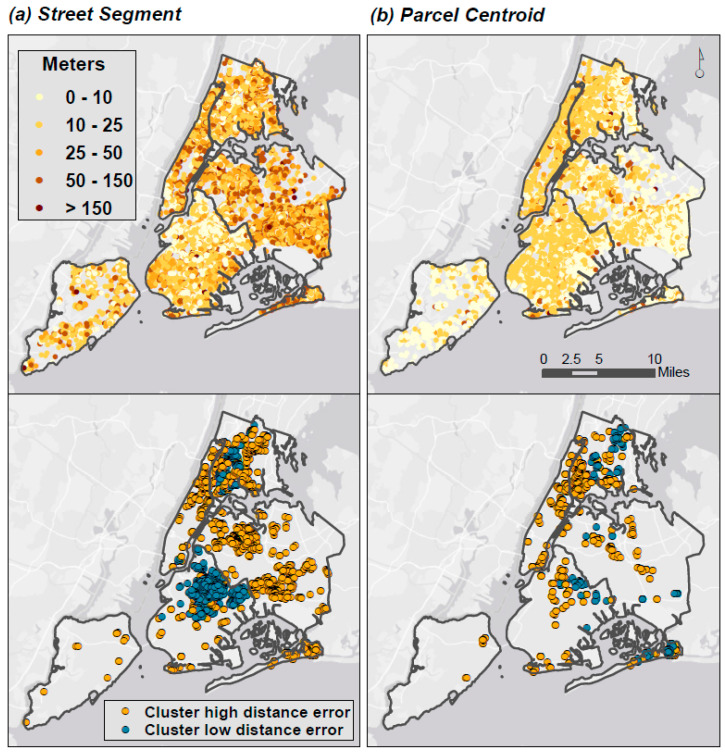
Spatial patterns of distance error between methods. Maps of (**a**) spatial pattern of distance errors (meters) and (**b**) clusters of high and low distance errors between Street Segment and Address Point geocodes, and between Parcel Centroid and Address Point geocodes. High clusters are points with longer distances between geocoding methods and low clusters are points with shorter distances between geocoding methods.

We found different spatial patterns in distance errors via the geocoding method, with relatively stronger clusters of high and low distance errors for Street Segment geocodes, and a relatively more dispersed error pattern for Parcel Centroid (Figure 3). For Street Segment geocodes, significant clusters of longer distance errors were seen in Manhattan and Queens, while smaller errors were clustered in Brooklyn and the Bronx. For Parcel Centroid geocodes, distance errors were smaller, on average, and more generally dispersed, though some clustering in both large and small distance errors were observed in these same neighborhoods.

### 3.3. Directional Error

**Figure 4:**
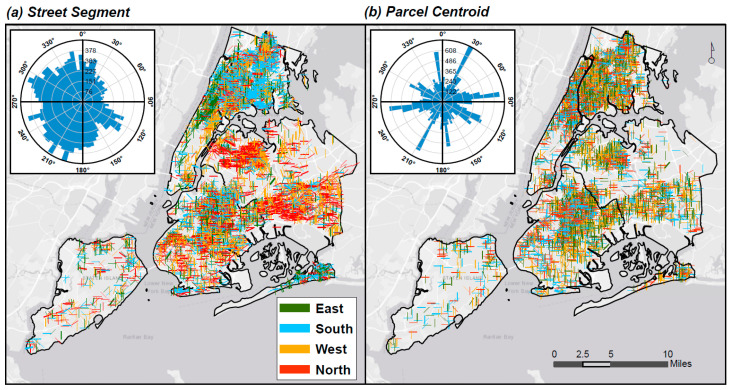
Spatial patterns of cardinal directional errors between methods. Maps of (**a**) Street Segment and (**b**) Parcel Centroid directional error compared to Address Points. Directional offset rose plots [49] show the number of observations by direction aggregated into 5-degree bins. Histograms show the distribution of compass angles by degree.

The angular differences in directional errors (i.e., direction from the Address Point (reference) geocode) were classified into the four cardinal directions for mapping purposes (Figure 4). We found more pronounced spatial patterning in directional errors for Street Segment than for Parcel Centroid geocodes. Street segment geocodes were systematically displaced to the north in parts of Queens, and to the south in northern Manhattan.

For Street Segment geocodes, overall, the mean vector direction of error was 250.8° (±1.87 degrees), and was systematically biased to the west of the reference location, as revealed in the rose plot (embedded in Figure 4a). Frequency counts of directional error, by the four compass directions, indicate that most Street Segment geocodes were displaced to the south or west (Table S2 in Supplemental Materials).

For Parcel Centroid geocodes, the mean vector direction of error was 91.5° (±8.2 degree) (i.e., on average, geocodes were displaced to the east of the reference location), though the rose plot revealed a less consistent and spiky directional trend (embedded in Figure 4b).

### 3.4. Impacts on Air Pollution Exposure Estimates

On average, the geocoding-attributable error in exposure assignments was small. NO_2_ was over-estimated by an average of 0.12 ppb (+0.56%) using the Street Segment locator, and under-estimated by an average of 0.06 ppb (−0.15%) using the Parcel Centroid locator. Though small, these average errors in NO_2_ estimates were, in most cases, significantly larger than those for PM_2.5_ (+0.34% using Street Segment (*p* < 0.0001 for NO_2_-PM_2.5_ difference), −0.19% using Parcel Centroid (*p* = 0.06)) or for O_3_ (−0.11% using Street Segment (*p* < 0.0001), +0.13% using Parcel Centroid (t-test *p* < 0.0001)). This slightly greater impact of geocoding error for estimates of NO_2_ may be in keeping with the relatively finer spatial variation observed in NO_2_ relative to other pollutants in NYC.

Although average geocoding-attributable exposure misclassification was very small, the range was very large; in some locations, NO_2_ was under-estimated by as much as −13.1 ppb (−35.3%), or over-estimated by as much as 12.3 ppb (44.8%) using the Street Segment locator. The comparable range using the Parcel Centroid locator was −35.3% to 60.9%.

As expected, locations which poorly predicted NO_2_ also tended to poorly predict concentrations of PM_2.5_ and O_3_. Generally, the locations where NO_2_ was over-estimated also had over-estimates of PM_2.5_ (r = 0.58). Errors in both primary pollutants were negatively correlated with errors in estimated O_3_ (r = −0.60 for NO_2_, r = −0.33 for PM_2.5_), which is in keeping with the inverse spatial patterning of O_3_, a secondary pollutant, which has relatively lower concentrations in denser parts of NYC.

**Figure 5:**
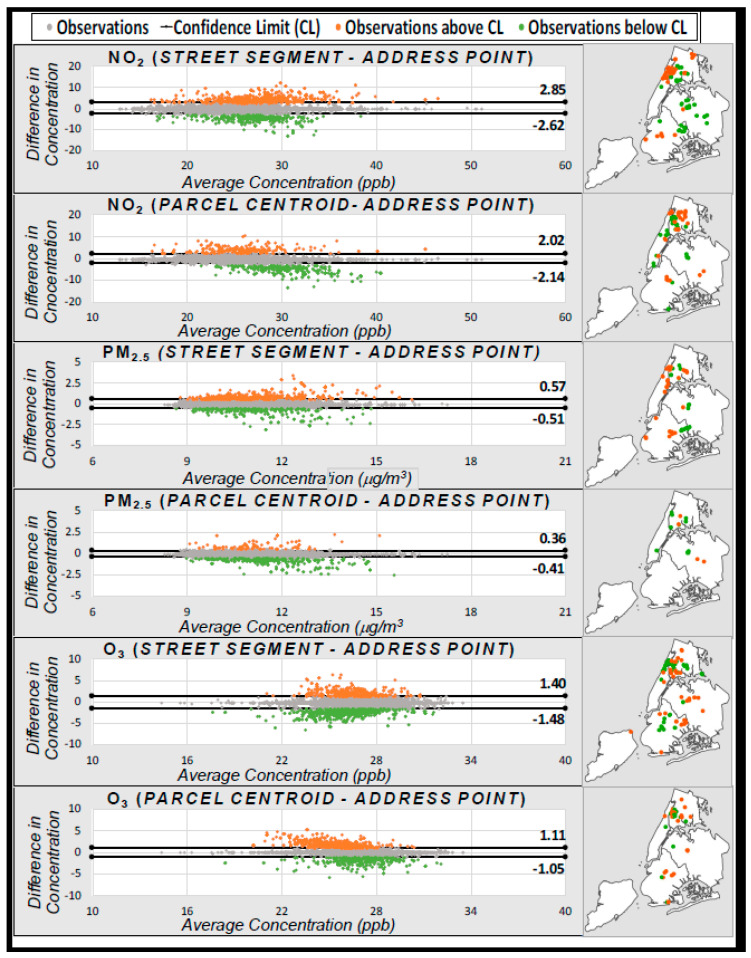
Bland–Altman plots of pollutant exposure misclassification. The x-axis depicts the average concentration estimate, based on the Address Point and alternative geocode; the y-axis depicts the difference in concentration estimate from the Address Point value (“error”) using the alternative geocode. Orange points on maps indicate significant clusters of over-estimates (above 95% Confidence Limit (CL)); green points indicate significant clusters of under-estimates (below 95% CL).

We examined the misclassification of estimated concentrations using Bland–Altman plots, which display the error in concentration estimates, as a function of the concentration itself (using the mean of concentrations derived from the reference (Address Point) and each alternative geocoding method) (*x*-axis) (Figure 5). We observed some evidence of differential misclassification, with greater misclassification where there were higher concentrations of both NO_2_ and PM_2.5_ (i.e., a funnel-shaped wider dispersion of errors reading left to right along the *x*-axis). The opposite was true for O_3_.

Although the average observed error was near zero for both methods, there were distinct outliers; using Street Segment geocodes, 6.5% of the points fell outside the 95% Confidence Limits (which were −2.62 to +2.85 ppb), while using Parcel Centroid geocodes, only 4.8% fell outside these limits (−2.14 to −2.02 ppb). Statistically-significant clusters of outliers were identified using Anselin Local Moran’s *I* and mapped (Figure 5, right).

### 3.5. Impacts on SEP Indicator Assignment

#### 3.5.1. Census Area Assignment

We found very few differences between geocoding methods in assignment to Census areas, even using Census blocks, the smallest Census area (Table S3 in Supplemental Materials).

#### 3.5.2. Differential Accuracy in Pollution Exposure Estimates by SEP

**Figure 6:**
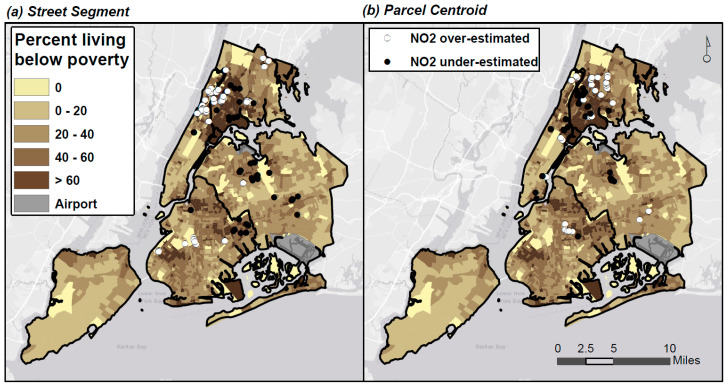
Spatial clusters of over- and under-estimates of NO_2_, by Census Tract poverty rates.

By overlaying the points for significantly over- and under-estimated NO_2_ exposures on Census Tract poverty rates (Figure 6), we observed substantial clustering in both over- and under-estimates in some lower-income parts of NYC (i.e., northern Manhattan/Bronx), using either Street Segment or Parcel Centroid methods. In addition, we observed some smaller clusters of over- or under-estimates in other parts of the city, which differed by geocoding method.

On average, we found greater pollution exposure misclassification in lower-SEP areas, especially using the Parcel Centroid locator. For example, in areas with clusters of over- or under-estimated NO_2_ (identified using the Bland–Altman plots in Figure 5), the average percentage of residents living below the poverty line was 28.1% (Table 1), which is marginally higher than the mean poverty rate of 26.1% for all geocoded addresses, but substantially higher than the mean poverty rate in NYC of 19.1%.

We compared distance error in ZIP code of above- vs. below-median number of addresses in the dataset, which roughly corresponds to address density in NYC, and found no differences in distance error by address density (Table S4 in Supplemental Materials).

### 3.6. Geocoding Uncertainty Surfaces

**Figure 7:**
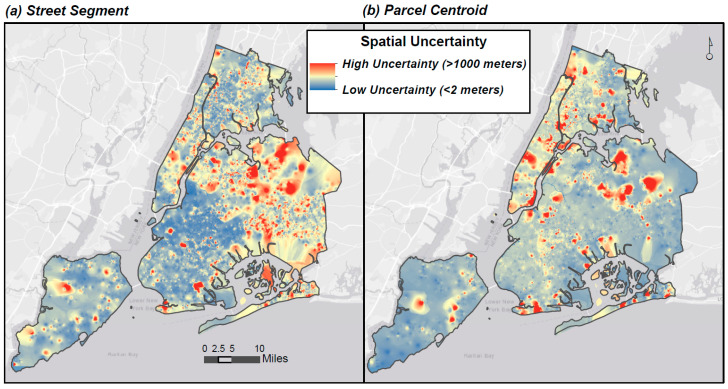
Geocoding spatial uncertainty surfaces, generated by interpolation of measured distance errors.

Surfaces of estimated distance errors (Figure 7) were developed to visualize expected geocoding error across different parts of the city, and to assess the potential uncertainty in exposure assignments. The surface values vary along a continuous scale of high to low distance errors, representing the degree of spatial uncertainty in locations across the domain.

Surface values are interpolated and cannot be used to extract exact distance errors. However, comparing actual distance error to the surface of ‘expected’ errors, for the Street Segment locator, indicated that, on average, the actual and predicted errors differed by less than 2 m (mean = 1.5 m), and fewer than 5% of the points had errors that differed by more than 25 m. For Parcel Centroid points, the average difference between actual and predicted error was only 0.6 m, and fewer than 0.01% had differences in errors of more than 25 m.

## 4. Discussion

Here, we presented and discussed broadly applicable methods for quantifying geocoding error, spatial patterning therein, and the implications of misclassification in air pollution exposures and SEP for environmental epidemiology studies. The methods and results presented in this paper have implications for methods and results reporting in environmental epidemiology studies that use geocoding methods for exposure assignment.

We found greater missingness using the Parcel Centroid vs. the Address Point or Street Segment locator, and significant spatial clustering in missingness, with patterns differing by method. The distance error from the Address Point was greater, on average, for Street Segment than for Parcel Centroid geocodes; notably, there were no distance errors of zero, and each method generated a different point for all 21,183 addresses. Error in Street Segment geocodes was highly clustered, and relatively more dispersed for Parcel Centroids. The spatial patterning in directional errors was more pronounced for Street Segment than for Parcel Centroid geocodes. Geocoding-attributable errors in pollution exposure estimates were small on average, but varied greatly, with some errors of 40% or more, and greater misclassification by percent with higher concentrations of NO_2_ or PM_2.5_. Geocoding errors did not influence Census Tract assignment, but the poverty rates were somewhat higher than the city average in the same areas where NO_2_ was systematically over-/under-estimated.

Taken together, our results demonstrate that multiple types of geocoding error, and spatial patterning therein, may lead to systematic missingness and/or over-/under-estimation of pollution exposures, with potential implications for health effect estimates, particularly in dense urban areas where exposures vary at a fine spatial scale. Given widespread reliance on geocoding for exposure assignment in environmental epidemiology, our results point to the need for the careful selection and consideration of geocoding methods that are used, and the thorough reporting and assessment of geocoding methods and potential consequential biases. Identifying ways to quantify and report spatial uncertainty is an ongoing challenge in geospatial analysis [57,58], as the magnitude and direction of errors can be propagated in spatial analyses wherein multiple spatial datasets are compared (e.g., geocodes vs. multiple pollutant exposure surfaces) [59].

We found that patterning in missingness differed by geocoding method, as expected given each method’s very different requirements. Street Address locators, for example, require addresses in a standardized format, and each precisely match a known address (i.e., in the exhaustive USPS catalogue of U.S. mailing addresses) [60]. In contrast, the high missingness rate we found for Parcel Centroid geocodes, also found in other studies (e.g., [15]), may be particularly high in NYC due to high rental rates, given that tax parcel databases often list the parcel owner’s (i.e., building owner) address, rather than the residential address on the parcel itself.

Distance errors in NYC averaged less than 30 m, but ranged up to several hundred meters, as elsewhere [8,17,19,61,62]. In dense urban areas, where pollution varies at a very fine scale among dense urban sources, [63] errors of this magnitude could substantially alter exposure estimates. In our analysis, positional errors led to few changes in SEP assignment using any level of Census geography, though prior studies have found impacts [24,64]. In NYC, the average Census block is very small (216.7 x 91.4 m), larger in both directions than our mean distance errors, resulting in few changes to point-in-polygon overlays. Studies in other areas have shown that the majority of geocoded points fall within the correct Census tract [45] (larger than Census blocks), and differences have been observed when assigning social exposures using fine-scale surfaces (rather than polygon areas), as have been created for assessing neighborhood disorder in several U.S. cities [65].

The directional displacement varied by method, and generally may influence pollution exposure estimates if the error follows the orientation of the key sources (i.e., roadways). The prevailing winds in NYC are generally from the west; as such, points displaced to the east of major roads may have over-estimated concentrations, and vice-versa. In our analysis, Street Segment points displayed a strong east–west displacement, likely due to their reliance on imputation along the NYC street grid (i.e., longer east–west blocks along streets, and shorter north–south blocks along avenues). The spiky, opposing peaks of the Parcel Centroid directional errors (embedded in Figure 4b) may be a product of small narrow lots in NYC, within which the centroid is set substantially back from the road, with little space to vary on either side.

Spatial uncertainty surfaces of geocoding distance errors were generated using IDW, which, by interpolating between points, assumes that locations near each other are more similar. Though interpolation can be problematic in dense areas with high local variability [66,67], IDW has compared favorably to other interpolation methods in that setting [68].

### 4.1. Strengths

Our study offered several strengths, including the use of a large, dense set of addresses from exhaustive hospital data including all NYC communities. The data size and density enabled us to examine spatial patterns and clustering in missingness and geocoding error in a more refined way than has previously been possible. Similarly, our use of very fine-scale air pollution surfaces and small Census areas in a dense urban setting enabled assessment of resultant errors in exposure classification via both environmental and social variables. Given the very small size of the Census areas in NYC, and our result that few Census area assignments were changed due to geocoding error, we can conclude that geocoding error may be unlikely to impact Census area assignments in most other locations, though it may have important bearing on the assessment of air pollution or other exposures with fine-scale variance.

Our methods—including the use of multiple location-specific geolocators—are fully adaptable to other study areas, allowing for the comparison of geocoding accuracy across study areas by method, and comparison between study areas in the relative improvements attributable to each method. Our use of Bland–Altman plots to examine exposure misclassification as a function of pollutant concentrations offers a means of quantifying differential misclassification likely to impact epidemiologic effect estimates. Finally, our use of IDW methods to generate spatial uncertainty surfaces provides a method for estimating the reliability and relative uncertainty of geocoding results across a study domain, and a means for identifying those locations where geocodes and resultant exposure estimates may be more or less precise.

### 4.2. Limitations

Nevertheless, our study does have several limitations. First, although NYC provides a very dense, SEP-diverse context in which to develop and validate these methods, it is also somewhat unique in those regards, and in its highly-gridded street structure and prevalence of multi-family housing units; all of these characteristics can influence geocoding accuracy, and thus our findings need be replicated elsewhere to establish generalizability [69].

In our study, we assessed missingness by ZIP Code, by virtue of needing a large enough area to calculate rates of missing addresses with variance, which would provide adequate variation in the numbers of missing addresses across the study area. ZIP codes, however, are too large to capture fine-scale variation, and thus there remains residual spatial variation in missingness. In addition, our addresses do not necessarily represent a random sample across all NYC areas (some ZIP codes are over-represented), with potential implications for generalizability.

Our use of Bland–Altman plots, commonly used to evaluate differential measurement bias [55,70], offered a means of quantifying and visualizing the relationship between exposure misclassification and pollutant concentrations—to determine whether such misclassification is systematic, or non-differential. These plots, however, and the 95% agreement intervals contained therein, do not determine whether the agreements between geocoding measures are acceptable; this must be defined within the context of the study parameters based on research considerations.

Our use of inverse distance weighting (IDW) to generate uncertainty surfaces is not without limitations. Though IDW has some advantages over other interpolation methods in urban settings [68], all interpolation methods risk “glossing over” important fine-scale variations in source-dense urban areas with substantial local variability. IDW, as an “exact interpolator,” assumes that all measurements are precise representations of that x,y location, and therefore fixes values in the IDW surface to exactly match each observation. Thus, local minima and maxima (inflection points) can only occur at sample points, and the resultant surface is highly sensitive to outliers and measurement error. The surface also depends strongly on selection of power value (*p*) and “search” strategy (selection of neighboring units used to generate each estimate), and thus care must be taken in setting these parameters.

Finally, as is the case for all analyses relying on the geocoding of residential addresses for exposure assignment purposes, these methods do not account for individual mobility, or exposures occurring outside of one’s residential area. Instead, residence-based exposure estimates are believed to capture a small but consistent portion of one’s exposure profile, generally representing chronic exposures over multiple years, and are useful as a means of comparing relative exposures across an urban cohort.

### 4.3. Suggestions/Recommendations

In environmental epidemiology or exposure science, geocoding methods are not always reported in the presentation of study methods. Given that we found substantial differences in missingness and accuracy using multiple geocoding methods—and, importantly, that many of these errors were differential with respect to pollutant concentrations—we suggest reporting the type of geocoding that was used as a common practice. Ideally, studies would also report summary statistics on missingness (match rate), preferably by sub-areas within the study domain, and specify the criteria used to generate that match rate (e.g., criterion used to determine acceptable accuracy in the spelling of street names).

Often, of course, the selection of geocoding method is a matter of practicality; Address Points are not yet available everywhere in the U.S., though Street Segments with address ranges are collected and maintained by the U.S. Census Bureau as part of the decennial Census, and are widely distributed online. Where possible, it may be preferable to use and compare multiple geocoding methods. It would also be useful to provide summary statistics on distance and directional error for each, or a map depicting which method was used to geocode each address, so as to identify systematic differences in methods across the study area. Investigators who obtain administrative health data that are already geocoded (e.g., vital statistics or electronic health record data) should be aware of the different methods for geocoding and the implications of these methods for exposure misclassification; when using pre-geocoded data, investigators should request detailed information on the geocoding methods used and details related to geocoding errors and missingness.

We suggest that epidemiologic studies explore spatial patterns in missingness and error, and assess systematic missingness and differential misclassification to the extent possible. Missingness may induce substantial biases by excluding certain types of neighborhoods in the study area (e.g., by excluding addresses with hyphenated street numbers or apartment numbers), or clusters of missingness may fall in particularly high- or low-pollution parts of the city. Optimally, studies should explore and report on systematic missingness and misclassification with respect to both exposures and outcomes of interest.

Simple sensitivity tests on epidemiologic results may include incorporating a geocoding accuracy score in multivariate models [26], or sub-analyses in strata defined by geocoding type, by strata of accuracy (i.e., using a geocoding score) or by sub-area percent missingness. Some researchers have incorporated local uncertainty in exposure measurement into regression models [71], or used mean values in a small diameter (buffer) around the geocode, rather than single points, to assign exposure estimates [72] to mitigate potential biases related to misspecification. Finally, in at least one case, researchers have used population weighted geo-imputation procedures to reduce missingness, using observed associations between PM_2.5_ and respiratory hospitalizations to assign unmatched geocodes to geographic locations [9].

Exposure scientists and epidemiologists may consider developing uncertainty surfaces and comparing these to observed spatial variation in the exposure of interest. Such analyses would inform on whether small variations in geocoding could plausibly alter exposure estimates in meaningful ways, or whether observations are differentially missing in high- or low-exposure parts of the study area.

## 5. Conclusions

We quantified and compared missingness, distance and directional error, and spatial patterns therein, across three geocoding methods, and quantified the impact of each on misclassification in air pollution and Census-based sociodemographic variables, across NYC. Our findings reveal substantial differences by geocoding methods, and evidence of differential misclassification, with greater geocoding-attributable errors in areas of higher NO_2_ and PM_2.5_ concentrations.

Our results highlight the importance of assessing spatial patterning in errors, particularly in dense urban settings, and the utility of spatial analysis in identifying potential misclassification in exposure estimates and, consequently, epidemiologic effect estimates. The methods developed and presented here also provide a means for the sensitivity analysis of epidemiologic results based on spatially-derived exposure estimates. Further study is needed to examine how geocoding error may be related to underlying spatial and non-spatial processes (e.g., address format, street structure, building density) that may influence geocoding accuracy.

Our findings demonstrate that geocoding method selection may alter exposure assignments—potentially systematically, with respect to exposure estimates. As such, our results support reporting on geocoding methods in epidemiologic studies, with attention to differential missingness and error across the study area.

## Figures and Tables

**Table 1:** Mean NO_2_ (ppb) and poverty rate for over- and under-estimated NO_2_ exposure points *.

Error Type	Frequency (*%*)	Mean NO_2_ (*+/− SD*) (ppb)	Mean Percent Below Federal Poverty Level (*+/− SD*)
Street Segment geocodes over-estimating NO_2_	855 (4.0%)	30.4 ± 4.18	25.9 ± 11.5
Street Segment geocodes under-estimating NO_2_	519 (2.5%)	24.8 ± 3.0	26.9 ± 12.8
Parcel Centroid geocodes over-estimating NO_2_	426 (2.0%)	28.9 ± 4.3	28.6 ± 12.2
Parcel Centroid geocodes under-estimating NO_2_	584 (2.8%)	25.5 ± 3.0	30.9 ± 12.6
Total Frequency (*%*) Average (*+/−SD*)	2384 (11.3%)	25.6 ± 3.2	28.1 ± 12.3

* Over- and under-estimated points include all points that are outside the Bland–Altman Confidence Limits.

**Figure A1:**
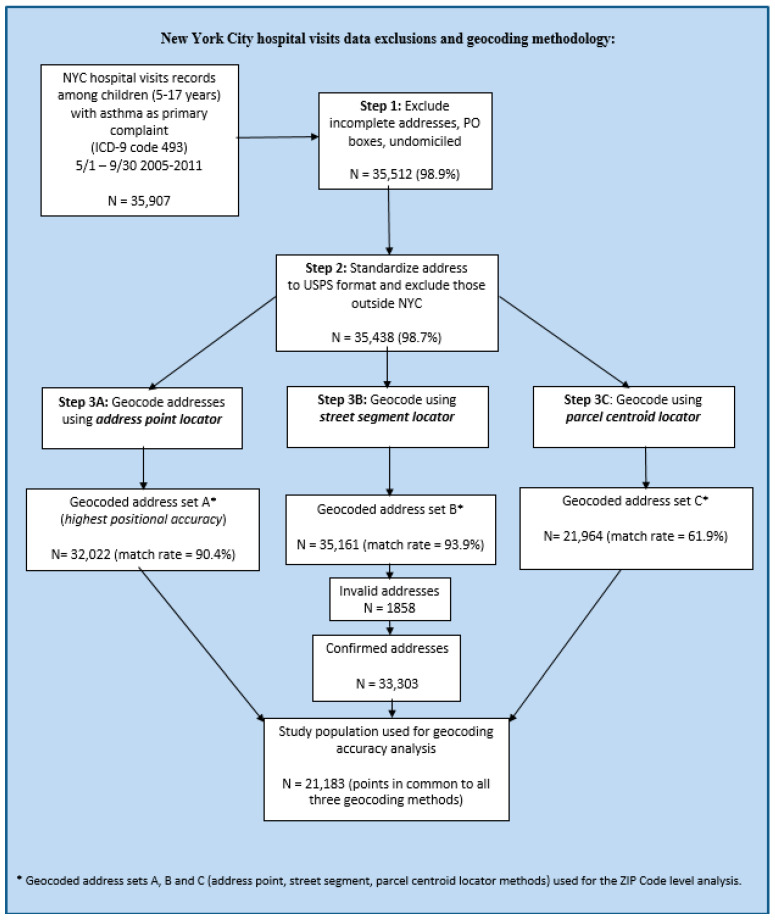
Address exclusions flow chart.

**Figure A2:**
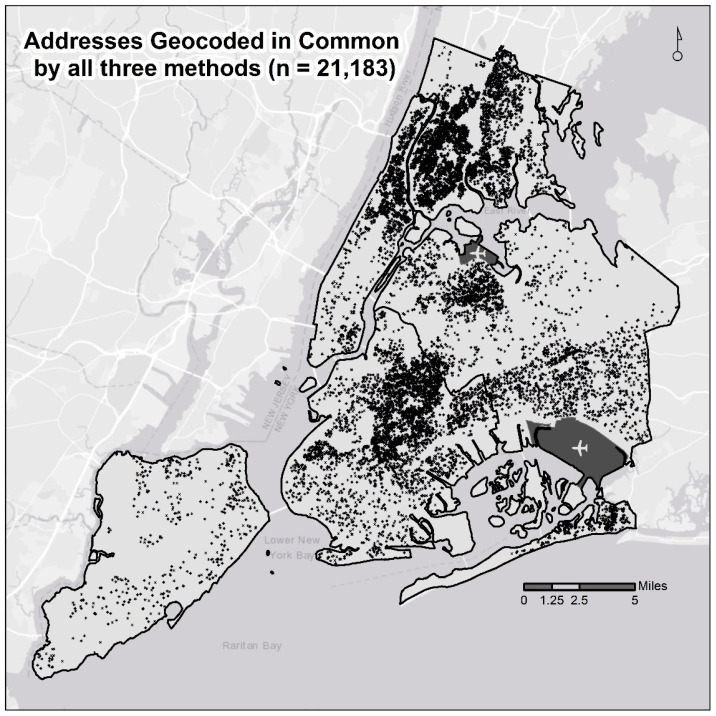
Spatial distribution of the resultant 21,183 residential addresses, successfully geocoded by all three methods, and used in this analysis.

## Supplementary Materials

The following are available online at https://www.mdpi.com/1660-4601/17/16/5845/s1, Figure S1: Street Segment geocodes using various side offsets, and distance error; Figure S2: Buffer-based exposure assignment for NO_2_; Figure S3: Repeat addresses in the dataset; Figure S4: Color-coded spatial uncertainty (distance error), overlaid with above/below-median number of addresses per ZIP code; Table S1: ZIP Code-level missingness rates; Table S2: Frequency (percentage) with directional error in each of four directional quadrants; Table S3: Change in Census Tract, Block Group, and Block assignment.

## Appendix A. Detailed Geocoding Methods

Our geocoding process relies on a database of addresses—which we first clean and standardize to conform to United States Postal Service (USPS) address requirements—and an address locator, which specifies reference data and defines the rules for translating street addresses into geospatial data (i.e., assigning longitude and latitude coordinates). The choice of address locator involves implicit trade-offs between maximizing the match rate (increasing the statistical power of the resultant analytic dataset) versus positional accuracy (minimizing exposure misclassification). Our geocoding processing aims are to (1) maximize the address match rate within each locator by cleaning and standardizing address data, and (2) utilize accurate, readily-available reference data that is contemporaneous with our address data.

All geocoding was performed offline to preserve data integrity and address confidentiality.

### Appendix A.1. Address Cleaning

We first excluded all incomplete address records (e.g., those having no street number), those outside of the five boroughs of NYC (by zip codes), and P.O. Boxes. Records coded for homeless hospital patients (e.g., 999 (missing); homeless; undomiciled) were excluded.

Patient address data is collected by individual hospitals, often using different practices, and frequently needs substantial format standardization to meet U.S. Postal Service specifications, and be compatible with GIS-based address locators. To avoid spatially systematic geocoding errors related to hospital-specific practices for address entry, or related to local differences in address structures (e.g., Queens uses a distinct address format of hyphenated street numbers), we standardized common address abbreviations (e.g., BLVD for Boulevard) and applied ZP4™ address standardization software (Semaphore Corporation, Monterey, CA, USA), using U.S. Postal Service reference databases to correct and standardize address format. Data cleaning was performed in SAS™ (SAS Institute, Cary, NC, USA) and gVim 7.4 (Softonic, Barcelona Spain), an open source PC version of the Unix vi editor. See address exclusions in Figure A1 below.

### Appendix A.2. Address Locators

All geocoding was performed in ArcGIS 10.2 using custom address locators, configured to maximize match rates and performed offline to ensure patient confidentiality.

Three types of address locators were used: Address Points, Street Locators and Parcel Centroid.

1. The *Address Point locator* is based on USPS postal delivery point locations, which allows for precise assignment of latitude and longitude coordinates at the front door, or mail delivery point, of individual building footprints or parcels.
  This layer provides the highest degree of positional accuracy for residential exposure assignment, but is the most restrictive, in that it requires complete and exact address formatting.
2. The *Parcel Centroid locator* matches each address to the corresponding tax Parcel Centroid, based on NYC tax parcel boundaries as determined by the NYC Department of Finance.
  Once the locator matches an address to a tax parcel, the geocode is assigned a latitude and longitude corresponding to the Parcel Centroid. This method provides a precise match with the information in tax records, but—because parcel lots vary widely with respect to size and buildings configuration—the positional accuracy of geocoded points varies substantially.
3. The *Street Segment locator* matches addresses to Street Segments, assigning coordinates based on the interpolated position of the address number along the Street Segment address range.
  The locator first matches address records by street name, and then uses the address number range to interpolate an address point location along the appropriate side of the street center line (i.e., even- vs. odd-numbered side of the street). For example, the address 1250 Manhattan Blvd. would be located at the approximate midpoint of the Street Segment with numeric range of 1200–1298 Manhattan Blvd., on the even-numbered side of the street. Positional accuracy is dependent on Street Segment length and building density. Because the addresses successfully matched in this layer are not validated against real-world address data (i.e., interpolated points along a Street Segment may be located on vacant lots, or the address may not exist in an official database (i.e., USPS deliverable addresses)), we used the ZP4 software to verify that these addresses were eligible for mail delivery and excluded any non-confirmed records.

### Appendix A.3. Side Offsets for Street Segment Locators

Side offsets are a pre-determined set distance used to shift geocoded points perpendicular to the road. Offsets are primarily used in Street Segment locators, to improve positional accuracy by moving points from the street centerline to the curb (i.e. closer to the building location). In contrast, the Address Point locator geocodes to the building front door or address delivery point, and the parcel point locator geocodes to the Parcel Centroid – both inherently located at a distance from the road.

The Dual Ranges Address style, used in this study, has a default side offset of 20 feet from the road, or a user-specified value can be applied. The same offset value is applied to all points, away from the road center line in the direction of the street side (odd vs. even) to which the point was originally matched. For this study, we used the default side offset value of 20 feet, which matches the average NYC street width of 40 feet, effectively moving geocoded points from the street centerline to a curb or sidewalk location, in front of the geocoded home.

We examined the impact of varying offset distances on the positional errors of street segment geocodes (compared to the address point reference geocodes). We found small improvements in accuracy for some locations, but these varied across the domain and, in some instances, distance errors were increased. Most of the distance errors in Street Segment geocodes are introduced by the interpolation which runs parallel along the Street Segment, rather than in slight shifts towards or away from the street centerline (Figure S1 in Supplemental Materials).

It is only possible to assign one offset distance for the entire set of addresses, which provides inconsistent positional improvements (as buildings vary in size and setback from the road, particularly for those in larger multi-building complexes). These positional improvements can be difficult to quantify, or may induce differential spatial error across the study domain (e.g., if larger multi-building complexes are differentially located in certain areas of the city). Several studies [8,45] found little or no improvement in measured errors as a result of using a consistent offset.

**Table A1:** Address locator settings.

Locator Setting	Address Point	Parcel Centroid	Street Segment
Style ^1^	US Address–Single House	US Address–Single House	US Address–Dual Ranges
Reference data	NYC Open Data Address Points https://data.cityofnewyork.us/City-Government/NYC-Address-Points/g6pj-hd8k	NYC Tax Parcel polygons http://www1.nyc.gov/site/planning/data-maps/open-data/dwn-pluto-mappluto.page	TeleAtlas StreetMaps Premium 2013 https://doc.arcgis.com/en/streetmap-premium/get-started/overview.htm
Minimum match score ^2^	85	85	85
Minimum candidate score ^2^	75	75	75
Spelling sensitivity ^2^	80	80	80
Side Offset ^2^	0	0	20

^1^https://desktop.arcgis.com/en/arcmap/10.5/manage-data/geocoding/commonly-used-address-locator-styles.htm^2^https://desktop.arcgis.com/en/arcmap/10.5/manage-data/geocoding/geocoding-options-properties.htm#ESRI_SECTION1_69547BEEDFC94FD78DE5A5D93A3CD195.
