# Supplementary material for: Geocoding Error, Spatial Uncertainty, and Implications for Exposure Assessment and Environmental Epidemiology
Source: Int J Environ Res Public Health. 2020 Aug 12;17(16):5845. doi: 10.3390/ijerph17165845 (PMC7459468; doi:10.3390/ijerph17165845)

Supplementary material for

Geocoding error, spatial uncertainty, and implications for exposure assessment and environmental epidemiology

Ellen J. Kinnee[1*], Sheila Tripathy[2], Leah Schinasi[3], Jessie L. C. Shmool[4], Perry E. Sheffield[5], Fernando Holguin[6], and Jane E. Clougherty[7]

[1] University Center for Social and Urban Research, University of Pittsburgh, Pittsburgh, Pennsylvania, United States ; ejk40@pitt.edu

[2] Drexel University Dornsife School of Public Health, Department of Environmental and Occupational Health, Philadelphia, Pennsylvania, United States; sheila.tripathy@gmail.com

[3] Drexel University Dornsife School of Public Health, Department of Environmental and Occupational Health, and Drexel University Urban Health Collaborative (UHC), Philadelphia, Pennsylvania, United States; lhs36@drexel.edu

[4] University of Pittsburgh Graduate School of Public Health, Department of Environmental and Occupational Health, Pittsburgh, Pennsylvania, United States; jlcshmool@gmail.com

[5] Environmental Medicine and Public Health and Pediatrics, Icahn School of Medicine at Mount Sinai, New York, New York United States; perry.sheffield@mssm.edu

[6] Department of Medicine, University of Colorado School of Medicine, Aurora, Colorado, United States

[7] Drexel University Dornsife School of Public Health, Department of Environmental and Occupational Health, Philadelphia, Pennsylvania, United States; jec373@drexel.edu

* Correspondence: ejk40@pitt.edu; Tel.: +1 412-385-5105

Figure S1: Street segment geocodes using various side offsets, and distance error. The default 20-foot offset (orange) is shown with calculated distance error from the reference (Address point). Alternative side offsets are shown [30 feet (blue), 40 feet (yellow), and 50 feet (purple)].

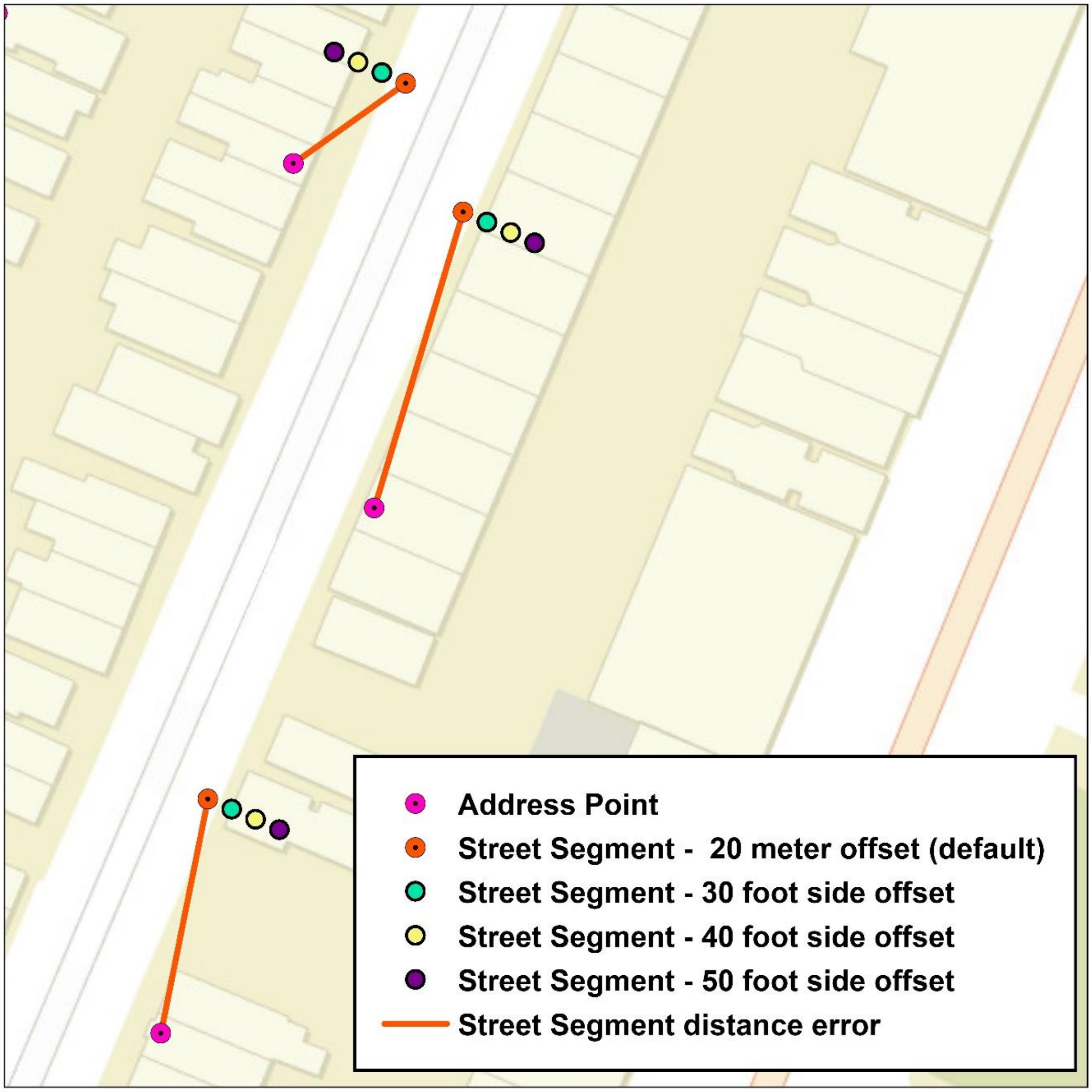

Figure S2: Buffer-based exposure assignment for NO_2, using each of the three geocoding methods and the NYCCAS NO_2 surface.

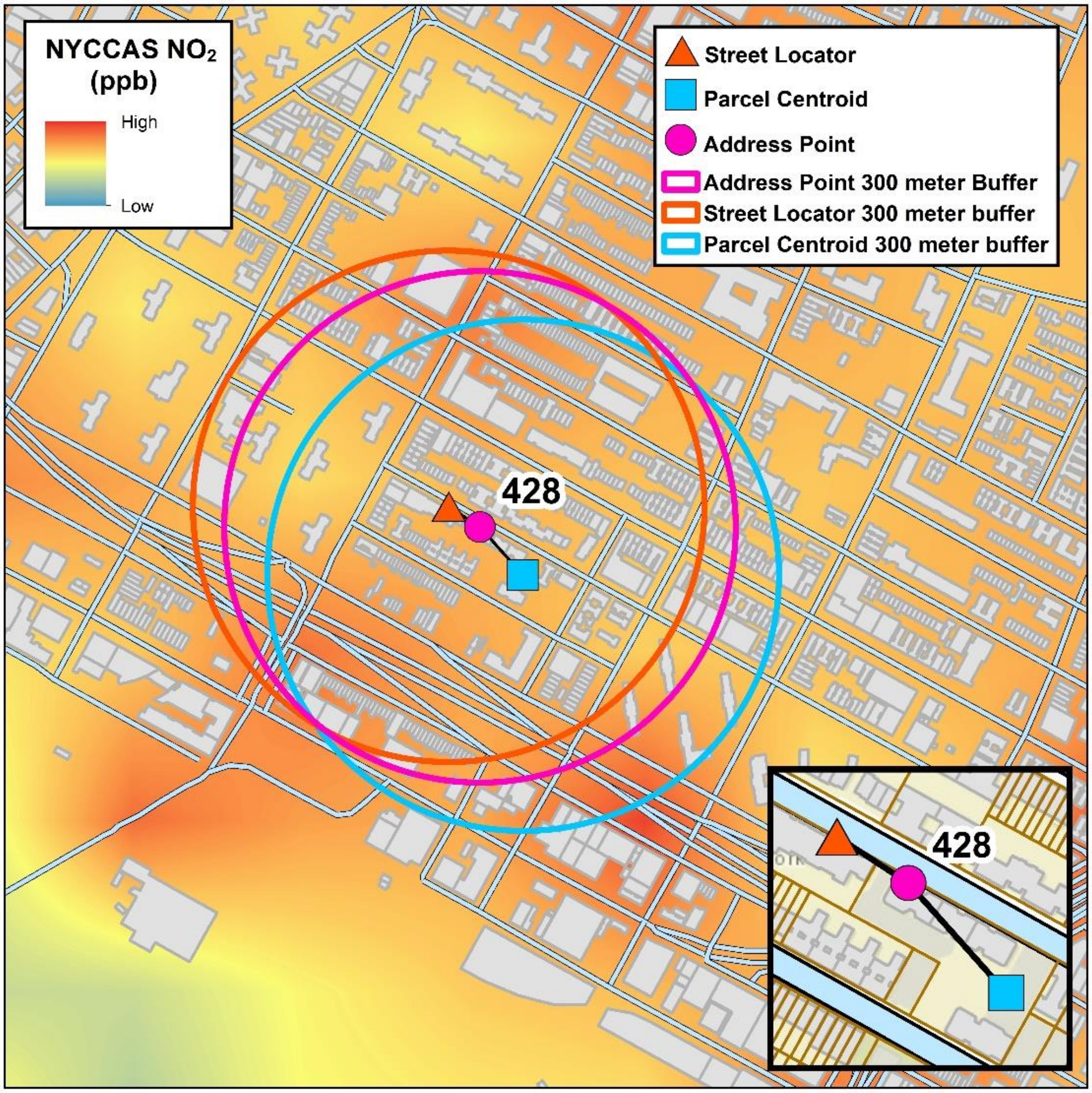

Figure S3: Repeat addresses in the dataset are shown in blue (1-5 obs/address), yellow (5-10 obs/address), or red (>10 obs/address).

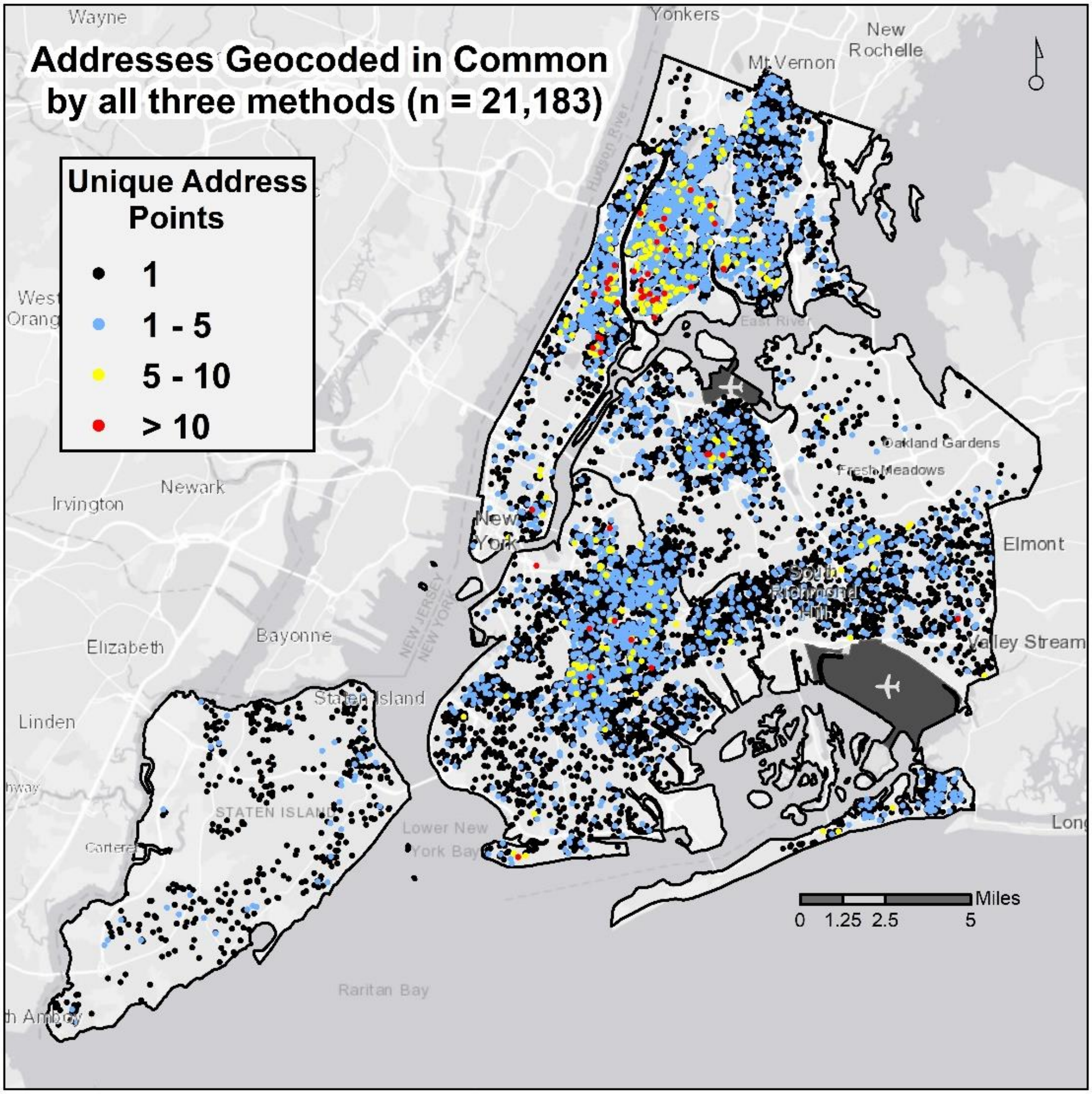

Table S1: Missingness rates: Overall missingness rate for all addresses (n=21,183), and by ZIP Code *(n= 157 ZIP Code areas)*

**Method (locator)**	**Overall missingness rate**	**Average missingness by ZIP**	**SD (by ZIP)**	**Median (by ZIP)**	**Min (by ZIP)**	**Max (by ZIP)**
**Address Point**	9.6	9.9	8.4	8.0	0	70.0
**Street Segment**	6.1	9.9	6.0	6.0	0	42.0
**Parcel Centroid**	38.1	35.0	23.5	28.0	0	93.0

Table S2: Frequency *(percentage)* with directional error in each of four directional quadrants, by method

**Method**	**North**	**East**	**South**	**West**
**Street Segment**	5,040 *(23.8%)*	3,853 *(18.2%)*	5,944 *(28.1%)*	6,346 *(29.9%)*
**Parcel Centroid**	4,968 *(23.5%)*	5,987 *(28.3%)*	4,836 *(22.8%)*	5,392 *(25.5%)*

Table S3: **Change in Census Tract, Block Group, and Block assignment** using Street Segment or Parcel Centroid geocodes, compared to Address points (n = 21,183)

Geocoding method	# Tracts changed (%)	# Block Groups changed (%)	# Blocks changed (%)
Street Segment	63 *(2.9%)*	188 *(1.2%)*	301 *(0.78%)*
Parcel Centroid	13 *(0.6%)*	119 *(0.77%)*	111 *(0.29%)*
Total in domain	2,167	15,464	38,794
Mean area of polygon (m^2)	362,888	129,780	20,186

Figure S4: Color-coded spatial uncertainty (distance error), overlaid with above/ below-median number of addresses per ZIP code. There is no consistent pattern in distance error by address density.

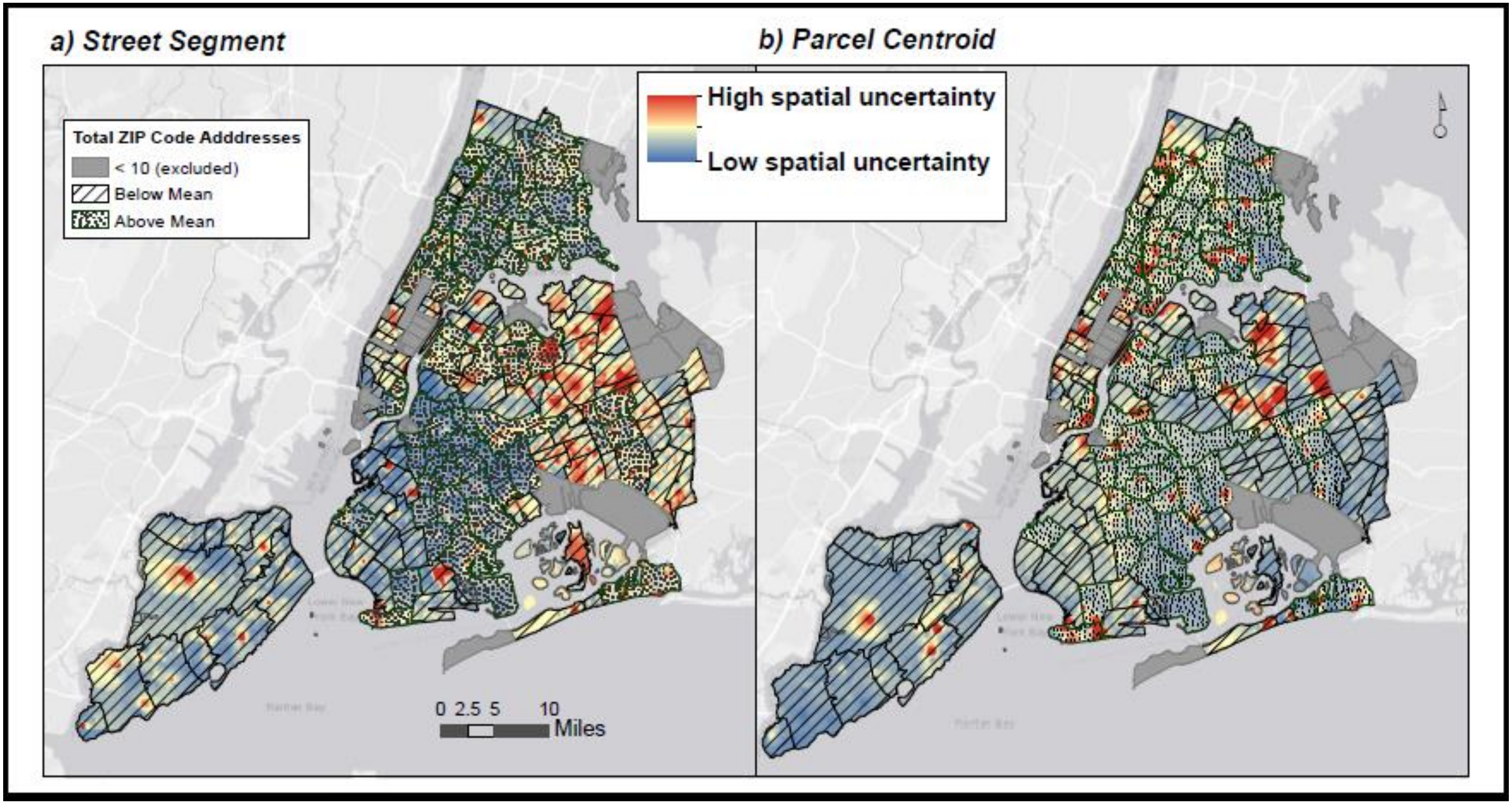

Supplement: Supplementary file 1 [file ijerph-17-05845-s001.pdf]
